# Compassion in Mexico and the United States: Unpacking Cultural Differences

**DOI:** 10.3390/bs15060732

**Published:** 2025-05-25

**Authors:** Naomi Hernandez, Liam Llerena, Evita Morales, Jack Tillman, David Ruiz Mendez, Birgit Koopmann-Holm

**Affiliations:** 1 Department of Psychology, Santa Clara University, Santa Clara, CA 95053, USA; 2 Facultad de Estudios Superiores Iztacala, Universidad Nacional Autónoma de México, Tlalnepantla 54090, Mexico; davidrm@iztacala.unam.mx

**Keywords:** compassion, culture, avoided negative affect, Mexico, United States

## Abstract

Previous research indicates that cultural variations exist in conceptualizations of compassion, potentially attributable to the extent to which individuals in diverse cultural settings want to avoid (versus accept) feeling negative emotions and the significance they place on emotion sharing as a component of compassion. The present study investigates the conceptualization of compassion among individuals in Mexico and the United States, aiming to understand why these cultural differences occur. We hypothesized that Mexicans (1) would want to avoid feeling negative less, (2) would consequently regard emotion sharing as a more critical element of a compassionate response, and (3) would therefore conceptualize a compassionate face as one that mirrors sadness more and expresses happiness less compared to U.S. Americans. Participants from Mexico and the United States engaged in a reverse correlation task, selecting stimuli that most closely resembled a compassionate face. The selected images were aggregated and coded for the extent of sadness and happiness depicted. Additionally, participants indicated how much they wanted to avoid feeling negative and, by using an open-ended format, described what a compassionate response would entail in their view. These responses were coded for whether or not they focused on emotion sharing. Consistent with our hypotheses, Mexicans, who want to avoid feeling negative less compared to U.S. Americans, place greater importance on emotion sharing in a compassionate response. This variation is associated with Mexicans conceptualizing a compassionate face as one that portrays more sadness and less happiness compared to U.S. Americans. People in different cultural contexts have different views about what compassion might entail. Understanding and embracing these cultural differences in compassion can help us navigate our increasingly multicultural world, fostering more meaningful connections and guiding our actions with more humility and sensitivity.

## 1. Introduction

In an era of increasing globalization and migration, societies are becoming more culturally, ethnically, and ideologically diverse. In this context, fostering mutual respect, understanding, and cooperation among members of society is more important than ever. To achieve this, compassion (i.e., the sensitivity to the suffering of others coupled with a motivation to alleviate the suffering; Goetz et al., 2010) is essential (e.g., Keltner, 2009). However, while people across cultures consider compassion to be important (Schwartz & Bardi, 2001), the ways in which compassion is conceptualized are far from uniform across cultures (e.g., Koopmann-Holm et al., 2021; Larco et al., 2024; Seow et al., 2025). These differences have real-world implications for how individuals interpret each other’s behaviors, build relationships across cultural lines, and navigate shared social spaces. If individuals interpret compassionate behavior through differing cultural lenses, miscommunication and misunderstanding may arise even in the context of well-intentioned actions. Conversely, recognizing and valuing the multiple ways compassion can be enacted may serve as a powerful bridge across cultural divides. In this article, we explore the cultural variability in conceptualizations of compassion, examine why it might occur, and discuss how an appreciation of this diversity can enhance mutual respect in multicultural societies. While past research has examined cultural differences in conceptualizations of compassion in contexts such as China, the U.S., and Germany (e.g., Koopmann-Holm et al., 2021; Seow et al., 2025), only one study has examined a Latin American cultural context (i.e., Ecuador; Larco et al., 2024). Clearly, more research on compassion in Latin America is needed.

The aim of the present research is to characterize what people in Mexico consider to be compassionate and to assess the extent to which the previously documented conceptualization of compassion identified in the United States can be replicated. Furthermore, we aim to understand why cultural differences in conceptualizations of compassion might occur between Mexico and the United States. To achieve these goals, participants in both cultural contexts selected stimuli that most resembled a compassionate face. To unpack cultural differences in conceptualizations of compassionate faces, participants also completed a measure of their desire to avoid feeling negative emotions, referred to as ‘Avoided Negative Affect‘ (ANA; Koopmann-Holm & Tsai, 2014). Additionally, to understand cultural differences in conceptualizations of a compassionate response, we asked participants to describe what they think a compassionate response entails. We then coded whether or not participants considered emotion sharing to be an important aspect. We examined whether ANA and the degree to which people consider emotion sharing to be a critical component of a compassionate response can explain Mexican–U.S. American cultural differences in conceptualizations of a compassionate face. Recognizing that compassion is conceptualized differently across cultures and understanding cultural differences in compassion can foster humility as people realize that what they consider to be compassionate might not necessarily be considered compassionate by others. With this understanding and humility, people can become truly compassionate and treat others the way *they* want to be treated.

### 1.1. Cultural Differences in Compassion

While compassion is defined as the sensitivity to another person’s suffering and motivation to relieve it (Goetz et al., 2010) and is valued by people across the world (Schwartz & Bardi, 2001), there is no single “compassionate response” or single compassionate facial expression (e.g., Falconer et al., 2019; Koopmann-Holm et al., 2021). Instead, individual and cultural differences exist in how people perceive and conceptualize compassionate reactions and facial expressions (Koopmann-Holm et al., 2021; Larco et al., 2024; Seow et al., 2025). For instance, imagine one of your acquaintances has lost a loved one and you want to show them that you care. You decide to send a sympathy card because you want to express your compassion. What type of sympathy card would you send? Similarly, which type of card would you consider to be more compassionate: one that focuses on the pain your acquaintance is feeling as well as on the loss and suffering (i.e., a card that focuses on the negative), or one that focuses on the silver lining (i.e., a card that focuses on the positive)? Past research has documented individual and cultural variation in what cards people feel more comfortable sending. People in the U.S. generally prefer to send sympathy cards that highlight uplifting messages rather than focusing on grief, whereas the opposite is true for people in Germany (Koopmann-Holm & Tsai, 2014). Similarly, these cultural differences are also seen in which types of cards people view as more compassionate: U.S. Americans consider cards that focus on the positive as more compassionate than negative cards, whereas Germans consider cards that focus on the negative as more compassionate than positive cards (Koopmann-Holm et al., 2021).

Do these cultural differences in sympathy card preferences extend to other forms of compassionate responses, such as the types of facial expressions people perceive as compassionate? Previous work suggests that people can differentiate between different forms of compassionate facial expressions, a kind compassionate facial expression, which indicates kindness with a slight smile, as well as an empathic compassionate facial expression, which signals emotion sharing by mirroring the distress of another person (Falconer et al., 2019). In line with the cultural differences in which sympathy cards people consider to be compassionate, for people in the U.S., a compassionate face depicts a small smile to show kindness and an emphasis on positive emotions (Koopmann-Holm et al., 2021; Seow et al., 2025). For people in China and Germany, a compassionate response entails the mirroring of the distress of others, emphasizing negative emotions (Koopmann-Holm et al., 2021; Seow et al., 2025). Past research on compassion has largely focused on WEIRD (Western, Educated, Industrialized, Rich, Democratic; Henrich et al., 2010) cultures (e.g., Koopmann-Holm et al., 2021) along with other commonly examined cultural settings such as China (e.g., Seow et al., 2025). However, one study exists that has explored how individuals from an underrepresented Latin American culture (i.e., Ecuador) conceptualize compassion (Larco et al., 2024), which we describe below.

### 1.2. The Case of Ecuador: Compassion Focusing on Both the Positive and the Negative

As outlined above, people in some cultural contexts (e.g., China and Germany) focus on the negative in their conceptualizations of compassion, while people in other cultural contexts (e.g., the U.S.) focus on the positive when conceptualizing compassion. More specifically, for people in China and Germany, a compassionate face depicts more negativity than positivity. In contrast, for U.S. Americans, a compassionate face depicts more positivity than negativity. Interestingly, very recently, a third form of compassion has been discovered, where people focus on both the positive and negative: While Ecuadorians conceptualize a compassionate face as depicting more sadness (i.e., mirroring the other person’s suffering) and less happiness compared to U.S. Americans, people in Ecuador conceptualize compassion as consisting of positive and negative aspects (Larco et al., 2024). That is, for them, a compassionate face depicts similar levels of positivity and negativity. These findings are in line with previous research suggesting that people in Ecuador and other Latin American cultural contexts highly value the positive (e.g., Senft et al., 2021) while also accepting the negative (e.g., Górriz et al., 2021a), which we review next.

#### 1.2.1. Valuing the Positive in Latin American Cultural Contexts

Latin American cultures are often characterized as collectivistic or interdependent, with an emphasis on in-group loyalty and close relationships with family and community members (Salvador et al., 2024). However, Latin American cultural contexts are different from East Asian cultural contexts, which are also described as collectivistic or interdependent (e.g., Kitayama et al., 2022). In Latin America, emotional expression is highly valued, which is not the case in East Asia (e.g., Salvador et al., 2024). Therefore, researchers use the term “emotionally expressive interdependence” (Salvador et al., 2024) to describe the different type of interdependence in Latin America. Other researchers argue that while Latin American societies are collectivist, they foster independent (rather than interdependent) selves, focusing on emotional expression (Krys et al., 2022a). A key cultural value that reflects this orientation toward emotional expression is *simpatía*, which involves the expression of positive emotions focused on relational warmth and affection (Senft et al., 2021). Through politeness and easygoingness, *simpatía* fosters high-quality social interactions and helps maintain harmony by steering away from behaviors that could create negativity or conflict (Acevedo et al., 2020; Senft et al., 2021).

Latin Americans, particularly Brazilians, have been stereotyped for their positive expressivity (Rezende, 2008). Indeed, research suggests that Brazilians exhibit higher levels of optimism compared to other cultural groups (de Oliveira & Nisbett, 2017). More broadly, Latin American societies as a whole tend to express high levels of positive emotions (Krys et al., 2022b). Additionally, Latin Americans tend to express socially engaging emotions that promote interdependence (Salvador et al., 2024), aligning with their collectivistic values. They also value positive affective states that are high in arousal, such as excitement and elation; an expression of these states might help foster harmonious interactions (Ruby et al., 2012).

While *simpatía* emphasizes the expression and valuation of positive emotions, it also involves avoiding negativity to preserve in-group harmony (e.g., Acevedo et al., 2020; Campos & Kim, 2017). This raises an important question: Does valuing the positive necessarily mean avoiding the negative? Affect Valuation Theory (Tsai et al., 2006) suggests that valuing positive emotions is not synonymous with wanting to avoid feeling negative emotions (Koopmann-Holm & Tsai, 2014). In fact, research indicates that the motivation to avoid negative affect is distinct from ideally wanting to feel positive affect (Koopmann-Holm & Tsai, 2014), suggesting that individuals can value positivity while also accepting negative emotions.

#### 1.2.2. Accepting the Negative in Latin American Cultural Contexts

Despite the emphasis on positivity in Latin American cultures and some past work suggesting that Latin Americans might avoid negativity (e.g., Acevedo et al., 2020; Campos & Kim, 2017), other research suggests that Latin Americans do not necessarily reject or devalue negative emotion expression (Senft et al., 2021). In fact, Latin American cultures encourage a broad range of emotional expression (Matsumoto et al., 2008; Murillo, 1976), and studies indicate that Mexican Americans report both more positive and more negative emotions compared to Chinese Americans (Soto et al., 2005). These findings regarding emotional expression and self-reported emotion suggest that Latin Americans might accept negative emotions as they balance the expression and experience of both positive and negative emotions.

In fact, concerning emotional experience, in the highlands of Ecuador, sadness and joy are not opposite states of mind (Tousignant, 1984). Other anthropological research conducted in Brazil (Da Matta, 1995) also suggests that things that appear to be opposites in some cultural contexts are not considered strict binaries in Latin America. For example, the death of a beloved person is a common source of negative emotions. However, at the burial of young children, Indigenous South American groups sing and dance with joy while crying simultaneously (Tousignant, 1984), suggesting that they may be more likely to not just express but also experience mixed (positive and negative) emotions.

Ethnographic studies of Ecuadorians further illustrate this acceptance of negative emotions. In Quito, which is located in the Ecuadorian Sierra, the emotion *pena* (akin to sorrow or affliction) is pervasive and plays a central role in emotional experiences (Tousignant, 1984; Tousignant & Maldonado, 1989). The prominence of *pena* in Quito suggests that Quiteños might be willing to accept feeling negative emotions. Moreover, unreciprocated feelings are a common cause of *pena* (Tousignant, 1984; Tousignant & Maldonado, 1989), highlighting the important role of emotion sharing and emotional reciprocity in Ecuadorian culture. Hence, for people in Ecuador, a compassionate response might consist of mirroring other people’s suffering. In support of this idea, social intimacy and empathy are greatly emphasized in Ecuador (Tousignant, 1984). For example, gathering with close members of one’s community leads to certain feelings of reciprocity (Tousignant & Maldonado, 1989). The importance of empathy and social continuity to people living in Quito is what makes them more aware of events where reciprocity may be lost. For example, offering a community member a meal and having this generosity rejected could lead to experiencing sadness and *pena* (Tousignant & Maldonado, 1989).

Empirical studies further support the idea that Latin Americans accept feeling negative emotions. For instance, Brazilians, compared to participants from the UK, reported being more accepting of emotional expression, particularly for negative emotions (Fonseca et al., 2023). In line with this, Latina mothers display a balance of both positive and negative reactions to their children’s emotions, while European American mothers respond with more positive expression (Lugo-Candelas et al., 2015). Similarly, Latin American children display more negative emotions compared to European American children (Lugo-Candelas et al., 2015). Regarding emotional experience, Ecuadorians report feeling more fear and sadness over the course of a month compared to people in Spain, a more individualistic culture (Górriz et al., 2021a). Importantly, retrospective reports of emotion over a long period might reflect generalized beliefs about emotion rather than actual emotional experiences (e.g., Robinson & Clore, 2002). Therefore, it is possible that Ecuadorians report feeling more negative emotions because they are not ashamed or afraid to report that they feel negative. In line with this, Ecuadorians also tend to observe, understand, and believe they can regulate their emotions more than Spaniards (Górriz et al., 2021b), suggesting a cultural inclination toward emotion awareness rather than avoidance.

Comparing Spain to the U.S., research suggests that Spanish participants experience extreme sadness, including crying, when feeling compassion for others (Schweiger et al., 2021). That is, Spanish participants conceptualize compassion as including negative emotions and crying. In contrast, U.S. Americans conceptualize compassion as entailing more positive emotions (Condon & Feldman Barrett, 2013; Shaver et al., 1987). Notably, Ecuadorians report even stronger negative emotions than Spaniards (Górriz et al., 2021a), suggesting that Ecuadorians might be even less inclined to avoid negative emotions than Spaniards and certainly U.S. Americans. Additionally, Ecuadorians engage in more emotion sharing when experiencing compassion (Chang et al., 2020), further reinforcing the idea that they accept and express distress rather than suppress it. All this work is in line with the finding that, compared to U.S. Americans, Ecuadorians conceptualize a compassionate face as one that depicts more sadness (i.e., mirrors the distress of a suffering person more) (Larco et al., 2024), even though Latin Americans also value positivity. Why do these cultural differences in compassion exist?

### 1.3. Unpacking Cultural Differences in Compassion: Avoided Negative Affect

Past research suggests that the previously reported cultural differences in compassion are related to the degree to which people want to avoid feeling negative emotions, termed ‘Avoided Negative Affect‘ (ANA; Koopmann-Holm & Tsai, 2014). Avoided Negative Affect is part of Affect Valuation Theory (AVT; Tsai et al., 2006), which differentiates actual affect (i.e., the affective states people actually feel) from ideal affect (i.e., the affective states people ideally want to feel) and avoided affect (i.e., the affective states people want to avoid feeling). Studies on affect and emotions typically assess the affective states that people actually feel (e.g., Brainerd et al., 2008; Chorpita et al., 1998; Forgas, 2008; Frasure-Smith et al., 1995; Gross & Levenson, 1997; Kiefer, 2005; Kuppens et al., 2008). In contrast to how frequently or intensely people actually feel certain affective states, ideal and avoided affect are affective goals. People want to be closer to the affective states they ideally want to feel and be further away from the states they want to avoid feeling. Prior research has shown that both ideal and avoided affect, which are influenced by cultural factors, can explain cultural differences in preferences and judgments, independent of actual affect (e.g., Koopmann-Holm et al., 2021; Seow et al., 2025; Tsai et al., 2006).

Interestingly, people in U.S. American cultural contexts want to avoid feeling negative more so than people in German, Chinese, and Ecuadorian cultural contexts do (e.g., Koopmann-Holm & Tsai, 2014; Koopmann-Holm et al., 2021; Larco et al., 2024; Seow et al., 2025). These findings are in line with other research suggesting that U.S. Americans consider negative emotions to be sinful and bad (Held & Bohart, 2002; Stearns, 1994) and that suffering is often transformed into stories of positivity (e.g., Canfield et al., 2009; Ehrenreich, 2009; McAdams, 2004). Germans, on the other hand, more openly express when something has gone badly (Hedderich, 1999) and are even considered melancholic and pessimistic (Clair, 2005; Gelfert, 2005). Similarly, people in East Asian (e.g., Miyamoto et al., 2014) and Latin American (e.g., Senft et al., 2021) cultural contexts accept negativity more than people in the U.S. do.

The motivation to avoid feeling negative is related to how people conceptualize compassion, i.e., the more people want to avoid feeling negative, the less they focus on the negative and the more they focus on the positive when responding with compassion. That is, when people want to avoid feeling negative to a great degree, instead of sending a sympathy card that focuses on the pain, they would rather send a card that focuses on the silver lining (Koopmann-Holm & Tsai, 2014). This allows them to avoid the negative, which is what they desire. These observations are consistent with the ‘avoided affect mismatch’ (Koopmann-Holm et al., 2020a), which suggests that ANA predicts how individuals respond when confronted with emotions they wish to avoid. Witnessing the death of a loved one is painful, and focusing on the positive, such as the silver lining, can help people with high levels of ANA to get away from what they want to avoid. For example, individuals who strongly wish to avoid negative emotions will frame their compassionate response to others’ suffering in a way that minimizes negativity, such as highlighting positive aspects of a situation or offering a warm smile.

Since U.S. Americans have a stronger desire to avoid negative emotions than Germans do, they also perceive sympathy cards and facial expressions that emphasize negativity as less comforting and compassionate compared to Germans (Koopmann-Holm et al., 2021). Likewise, because U.S. Americans are more inclined to avoid negative emotions than Chinese individuals, they view facial expressions conveying sadness as less compassionate than Chinese people do (Seow et al., 2025). Several studies have found that ANA functions as a mediator between culture and conceptualizations of compassion; that is, cultural differences in compassion can be explained by cultural differences in ANA.

Can ANA also explain the Ecuadorian–U.S. American cultural differences in compassion? One study sheds light on this issue, but this study did not just examine ANA as a mediator but also emotion sharing to understand the mechanism through which ANA might shape cultural differences in conceptualizations of a compassionate face (Larco et al., 2024), which we will now explore.

### 1.4. Unpacking Cultural Differences in Compassion Even Further: Emotion Sharing

How exactly does ANA influence how people conceptualize a compassionate face? Past research found initial support for the following sequential mediation model: ANA may shape how people conceptualize a compassionate face by influencing the importance they place on emotion sharing for a compassionate response (Larco et al., 2024). More specifically, those who want to avoid feeling negative emotions more are less likely to see emotion sharing as central to a compassionate response, because emotion sharing would imply sharing the negative emotions of the person who is suffering. This might lead them to think of a compassionate face as one that depicts more happiness and less sadness (Larco et al., 2024). Since Ecuadorians are less inclined to avoid negative emotions than U.S. Americans are, they consider emotion sharing to be more important for compassion and thus conceptualize a compassionate face as one that expresses more sadness and less happiness (Larco et al., 2024). This aligns with research showing that Latin Americans tend to feel more empathy (i.e., share the emotions others are feeling) when showing compassion than U.S. American and Turkish participants (Chang et al., 2020). It is important to examine individual and cultural differences in what people consider to be a central aspect of compassion, as past work has shown that some people might express compassion by sharing the emotions of the person who is suffering, whereas others might do so by expressing their desire to help (e.g., Goetz et al., 2010; Koopmann-Holm et al., 2020b; Ministero et al., 2018).

However, because the analyses examining this possible sequential mediation were only exploratory in previous research (Larco et al., 2024), more studies are needed to examine whether these relationships replicate. Furthermore, the findings that (1) Ecuadorians want to avoid feeling negative less than U.S. Americans and (2) for Ecuadorians, compassion focuses on both the positive *and* the negative might at first glance seem inconsistent with past research suggesting that people in Latin American cultural contexts focus on positivity (e.g., Senft et al., 2021) and avoid negativity (e.g., Acevedo et al., 2020; Campos & Kim, 2017; but see also studies that suggest that Latin Americans accept the negative, such as Górriz et al., 2021a). Because of this, we examined whether the cultural differences in ANA and compassion between Latin Americans and U.S. Americans would replicate in a new study, in which we included participants from a different Latin American cultural context, namely Mexico.

### 1.5. The Present Research: ANA, Emotion Sharing, and Compassionate Faces in Mexico

We predicted that the cultural differences between Ecuador and the U.S. would replicate in a new study with a different U.S. American and Latin American (i.e., Mexican) sample. To arrive at this prediction, we reviewed the literature about cultural similarities versus differences within Latin American cultural contexts, specifically focusing on similarities and differences between Ecuador and Mexico in emotion. Delgado et al. (2020) compared the frequency of usage of 100 Spanish emotion terms in the different Spanish-speaking areas of the world, including the Andean region (which includes Ecuador) and Mexico–Central America. Similar frequencies imply cross-cultural generalizability regarding the familiarity of emotion terms. Overall, they found evidence of consensus among these areas, suggesting that people in these different Spanish-speaking areas use certain emotion terms with similar frequencies. Hence, it is possible that the emotional lives of people in Ecuador and Mexico do not differ much.

Even though Delgado and colleagues did not specifically compare emotion terms related to compassion between these different areas, they report the frequencies of usage of different emotion terms for each region in two tables in their paper (see Tables 1 and 3; Delgado et al., 2020). Because *pena* plays a central role in Ecuador (Tousignant, 1984; Tousignant & Maldonado, 1989) and because Larco et al. (2024) derived their predictions regarding compassion and ANA in Ecuador partly from ethnographic work focusing on *pena*, we first examined whether the term *pena* is used with similar frequencies in the Andean region and in Mexico–Central America. The data in Table 1 in Delgado et al. (2020) suggest that the frequency of the use of *pena* in the Andean region is very similar to the frequency of the use of *pena* in Mexico. Furthermore, this table indicates that the frequency of the use of *pena* is much higher than the frequency of the use of any other emotion term in these regions. This suggests that for people in Mexico, similar to people in Ecuador, *pena* is a central emotion term, which in turn might influence people’s conceptualization of compassion. That is, it is possible that compassion is conceptualized similarly in Ecuador and Mexico.

We also carefully examined Table 3 in Delgado et al. (2020), which includes emotion terms that are similar to the concept of emotion sharing, namely *empatía* (which translates as empathy into English) and *conmiseración* (which translates as commiseration into English). Once again, these terms have similar frequencies in the Andean region and Mexico–Central America, suggesting that the centrality of emotion sharing (which includes empathy and commiseration) might be similar in Mexico and Ecuador. However, these terms had much lower frequencies in the U.S. compared to the Andean region and Mexico–Central America. Hence, emotion sharing seems to be less central in the U.S. compared to Latin America, which is in line with past research (Larco et al., 2024). Taken together, it is possible that Ecuador and Mexico might have similar conceptualizations of a compassionate face and similar conceptualizations of a compassionate response, as *pena* and emotion sharing seem central in both of these Latin American cultural contexts. Finally, Ecuador and Mexico seem to differ in these aspects from the U.S. to a similar degree.

We observed one additional interesting pattern across both Tables 1 and 3 in Delgado et al. (2020). In Table 1 in Delgado et al. (2020), the U.S. has the lowest frequency for eight out of the 31 negative emotion terms. Because Delgado et al. (2020) compared eight regions, we would expect that each region would score the lowest on 3.88 emotion terms due to chance (31/8), which is much lower than the observed frequency of eight for the U.S. Similarly, in Table 3 in Delgado et al. (2020), of the 25 negative terms, the U.S. had the lowest frequency for seven terms, a much higher frequency than the expected frequency of 3.13 (25/8). Both of these findings suggest that in the U.S., people use negative emotion terms less frequently than people in other regions, such as the Andean region and Mexico–Central America. Our prediction in the present study that people in the U.S. might want to avoid feeling negative more not only compared to people in Ecuador but also compared to people in Mexico is thus in line with Delgado et al.’s (2020) research. In fact, people within the cultural context of the U.S. seem to be particularly motivated to avoid using negative emotion terms, more so than people in seven different Spanish-speaking regions. Because of this, we predicted that the previously reported Ecuadorian–U.S. American cultural differences in ANA, emotion sharing, and compassion would replicate in another Latin American cultural context (i.e., Mexico).

Similar to the methodology used by Larco et al. (2024) to examine how Mexicans and U.S. Americans conceptualize a compassionate face, participants completed a reverse correlation task. This task investigates individuals’ mental representations of faces with various traits, such as dominance, trustworthiness, or compassion (e.g., Dotsch & Todorov, 2012; Koopmann-Holm et al., 2021). To understand why cultural differences in conceptualizations of compassion might exist, participants also completed a measure of ANA. Additionally, to explore the extent to which people associate emotion sharing with compassion, participants were asked to describe, in an open-ended format, the most compassionate response to someone who has recently lost a loved one. The responses were then coded for whether or not they mentioned emotion sharing. Beyond emotion sharing, we also assessed whether or not these responses mentioned expressions of love and kindness as another way to express compassion to examine whether our findings were specific to emotion sharing. We neither anticipated cultural differences in mean levels of expressions of love and kindness nor did we anticipate that expressions of love and kindness would be associated with conceptualizations of a compassionate face.

Scholars have recommended that future research on moral emotions such as compassion should be based on theory (Londoño-Cortés et al., 2023). The present research does exactly this. We derive our predictions about cultural differences in compassion from the theoretical framework of AVT (Tsai et al., 2006; for an extension of this theory, see Koopmann-Holm & Tsai, 2014).

We hypothesized that Mexicans would want to avoid feeling negative less than U.S. Americans would (Hypothesis 1). Furthermore, people who are less inclined to avoid feeling negative emotions may be more likely to view emotion sharing—such as expressing sadness alongside someone who is suffering—as a key part of a compassionate response. Because they are more open to sharing negative emotions, they may also associate compassion with facial expressions that show more sadness (as a sign of emotion sharing) and less happiness. Therefore, we also predicted that, similarly to Ecuadorians, for participants from Mexico, a compassionate response would consist of more emotion sharing (Hypothesis 2), and their mental representations of a compassionate face would depict more sadness (i.e., would mirror other people’s suffering more) and less happiness (i.e., less of a kind smile) compared to U.S. Americans’ mental representations (Hypothesis 3). The prediction about happiness might at first seem at odds with the fact that Latin Americans highly value positive emotions. However, while positive emotions are highly valued in Latin American cultural contexts, other research suggests that European Americans may value positive emotions even more compared to Latin Americans (Larco et al., 2024; Senft et al., 2021), which is in line with our predictions. Finally, we hypothesized a sequential mediation in which culture would be indirectly associated with conceptualizations of a compassionate face through ANA and conceptualizations of a compassionate response as emotion sharing (Hypothesis 4).

## 2. Method

### 2.1. Participants

We recruited participants from Santa Clara University in the United States and Universidad Nacional Autónoma de México in Mexico. The U.S. sample consisted of 59 participants (59.32% female, 38.98% male, 1.69% did not specify; mean age = 19.02 years, *SD* = 1.00; 61.02% White, 13.56% Latinx/Hispanic, 3.39% African/African American, 8.47% Mixed, 3.39% Middle Eastern, 10.17% did not specify), while the Mexican sample included 55 participants (67.27% female, 30.91% male, 1.82% did not specify; mean age = 22.30 years, *SD* = 2.85; 3.64% Mestizos, 9.09% Latinx, 14.55% Mexican, 72.73% did not specify—they often responded to the ethnicity question with “ninguno” (none); this may be because a majority of Mexico’s population (around 70%) identifies as Mestizo leading them to believe ethnicity is a term that refers only to minority groups such as Indigenous peoples). To be included in this study, Mexican participants had to be 18 years of age or older and had to be born and raised in Mexico. U.S. American participants had to be 18 years or older and had to be born and raised in the U.S. For their participation, U.S. American participants received course credit in their introductory psychology courses, while Mexican participants received a $5 Amazon gift card. All participants completed the measures online. U.S. American participants completed the measures listed below (in that order) in English, while Mexican participants completed them in the same order in Spanish. To ensure accuracy and equivalency, two independent bilingual research assistants used the translation–back translation technique. In addition to the measures reported below, we included a few other measures to collect pilot data for new projects.^1^

Sample size was determined using G*Power 3.1.9.7 software—ensuring a power of 0.80 to detect medium-size effects (*f* = 0.25) using an ANCOVA with two groups, at least one covariate, and an alpha error probability of 0.05. Therefore, we needed a total sample size of 128 participants. We aimed for 60 participants in each country; however, some participants clicked on the survey link but then did not complete the study, which is why we ended up with sample sizes approaching 60. We used the data from all participants who completed the survey.

The two groups did not differ in gender distribution (*χ*^2^ [2, 112] = 0.81, *p* = 0.37, Cramer’s V = 0.09). However, the two groups differed in self-reported socioeconomic status (*F* [1, 108] = 40.33, *p* < 0.001, *η_p_*^2^ = 0.27). On a scale ranging from low income (“1”) to high income (“5”), in the U.S., on average, participants reported to have an average (“3”) to an upper middle income (“4”); in Mexico, on average, participants reported to have a lower middle income (“2”) to an average income (“3”); U.S. Americans: *M* = 3.89, *SD* = 0.91; Mexicans: *M* = 2.74, *SD* = 0.99. Furthermore, Mexicans were significantly older than U.S. Americans (*F* [1, 110] = 68.02, *p* < 0.001, *η_p_*^2^ = 0.38). Therefore, we initially controlled for socioeconomic status and age in the analyses reported below, but because neither age nor socioeconomic status emerged as significant covariates, we removed them from our final analyses to conserve power. The pattern of findings did not change when we included socioeconomic status and age as covariates.

### 2.2. Materials and Procedure

#### 2.2.1. Reverse Correlation Task

We used Qualtrics to examine how Mexicans and U.S. Americans conceptualize a compassionate face. On a computer, participants completed a 2-image forced-choice reverse correlation task with 300 trials (e.g., Dotsch & Todorov, 2012). For each trial, participants were presented with a fixation cross for 1000 ms before they saw a pair of faces. For each face pair, participants were prompted to “please select the stimulus that most resembles a compassionate face” for U.S. participants and “*por favor seleccione el estímulo que más se parezca a una cara compasiva*” for Mexican participants. All participants selected one of the faces in each pair by clicking on the respective image.

Given that we had 300 trials for each participant, we were unable to randomize the order of all the trials without having the software malfunction. To solve the problem, we created 30 blocks with 10 image pairs each (e.g., block 1 contained image pairs 1–10, block 2 contained image pairs 11–20, and so forth). Because we randomized the order of each block, and the stimuli in the reverse correlation task were created with random noise, it was not necessary to randomly present the image pairs.

We used an existing R script package (rcicr R-Package; Dotsch, 2016; Dotsch & Todorov, 2012) in the open-source program R 4.3.1 (R Core Team, 2018) to create the stimuli and, after the completion of the study, to compute individual composite images (CIs) by averaging the selected noise patterns for each participant (individual CIs; Brinkman et al., 2017).

*Base Face and Creation of Stimuli.* The face pairs were created by using a base face that matched the majority of participants’ ethnicities in each cultural context. That is, for U.S. participants, we used a White base face. The base face consisted of a grayscale average of all the neutral female faces photographed without angles in the Averaged Karolinska Face Database (Lundqvist & Litton, 1998). For Mexican participants, we created a Latina base face by choosing all female faces from the Chicago Face Database (Ma et al., 2015) that were at least 60% Latina (percentages were calculated based on the number of participants who rated the face as Hispanic/Latin American divided by the total number of people who rated the face). These totaled 22 faces. Then, we used the free morphing software Sqirlz Morph to morph these 22 faces into the resulting base face. These same base faces were used in previous research (see Figure 2 in Larco et al., 2024).

Using the *rcicr* R-Package (Dotsch, 2016; Dotsch & Todorov, 2012), we superimposed random noise patterns onto the base faces. For each base face, we generated 300 pairs of faces, where one face in each pair had a unique random noise pattern overlaid, while the other featured the same base face with the inverse (i.e., each original white pixel is black, and each original black pixel is white) of this unique random noise pattern overlaid. These 300 face pairs were the same as used in a previous study (Larco et al., 2024).

#### 2.2.2. Aggregating and Coding of the Individual Composite Images

To generate individual composite images for each participant (which can be regarded as that person’s conceptualization of a compassionate face), we employed an existing R script package (rcicr package; Dotsch, 2016; Dotsch & Todorov, 2012) in the open-source program R (R Core Team, 2018) to average the selected noise patterns for each participant (individual composite images; Brinkman et al., 2017). Our analysis focuses on these individual composite images to maintain individual variation in participants’ responses (Cone et al., 2020).

Three research assistants (one female Latina student, who was born and raised in Mexico but lived in the U.S. during the time this research was conducted; one female White student, who was born and raised in the U.S.; and one male Latino student, who was born and raised in the U.S.) coded all resulting individual composite images. The coders rated each individual composite image on happiness and sadness using a three-point scale ranging from 0 (not present) to 1 (fully present). All coders were blind to the predictions and to the participants’ scores on other measures in our dataset. Interrater reliability was high for happiness (ranging from *r*(112) = 0.66, *p* < 0.001 to *r*(112) = 0.81, *p* < 0.001) and sadness (ranging from *r*(112) = 0.62, *p* < 0.001 to *r*(112) = 0.66, *p* < 0.001).

#### 2.2.3. Affect Valuation Index

Following the reverse correlation task, participants completed the extended version (Koopmann-Holm & Tsai, 2014) of the Affect Valuation Index (AVI; Tsai et al., 2006). The AVI assesses three types of affect, namely actual, ideal, and avoided affect (in that order), by asking participants to indicate how often they actually feel, how often they ideally want to feel, and how often they want to avoid feeling 37 affective states over the course of a typical week. Responses are recorded on a 5-point scale ranging from 1 (never) to 5 (all the time).

Past research suggests that mean-deviated scores should be used for AVI aggregates because there are cultural differences in how participants respond to the items of the AVI (e.g., Koopmann-Holm & Tsai, 2014). Furthermore, on average, people tend to differ in how much they want to avoid feeling emotions in general (e.g., Maio & Esses, 2001). Therefore, in line with past research (e.g., Koopmann-Holm & Tsai, 2014; Koopmann-Holm et al., 2021), we calculated mean-deviated scores by subtracting each participant’s overall average rating across the 37 avoided affect items from their raw score for each specific avoided affect item (e.g., avoided unhappy). The same procedure was applied to actual and ideal affect, where we subtracted the participant’s mean rating for all 37 actual (ideal) affect items from their raw score on each actual (ideal) affect item. Using these mean-deviated scores, we created aggregate scores for positive and negative affect. Again, in line with past research (e.g., Larco et al., 2024), for positive affect, we combined enthusiastic, excited, elated, happy, content, satisfied, calm, relaxed, and serene. For actual and ideal positive affect, internal consistencies (Cronbach’s alphas) were 0.85 and 0.80 for U.S. Americans and 0.83 and 0.76 for Mexicans, respectively. For negative affect, we combined sad, unhappy, lonely, fearful, hostile, nervous, dull, sleepy, and sluggish. For actual negative affect and ANA, Cronbach’s alphas were 0.84 and 0.94 for U.S. Americans and 0.86 and 0.86 for Mexicans, respectively.

#### 2.2.4. Coding of Description of Most Compassionate Response: Emotion Sharing and Expressions of Love/Kindness

Participants responded to the prompt “In your opinion, what is the most compassionate way that other people can respond to someone who has just lost a loved one? (Please describe below).” The participants wrote their responses into a text box. One female Latina research assistant, who was born and raised in Mexico but lived in the U.S. during the time the research was conducted; one female White U.S. American research assistant, who was born and raised in the U.S.; and one male Latino research assistant, who was born and raised in the U.S. (all fluent in Spanish and English), coded all English and Spanish open-ended responses. They coded whether or not participants consider emotion sharing to be part of a compassionate response to someone who is suffering by coding whether or not actively sharing experiences (e.g., relating to the person who is suffering, sharing the feelings of the person who is suffering) was part of the responses. The coders coded a response as “1” if the category was present and as “0” if the category was not present. Interrater reliability between the three coders was high (the percentage of agreement ranged from 95.50% to 98.20% with an average of 96.40%).

To examine whether our findings were specific to emotion sharing and not another form of expressing compassion (i.e., expressing love and kindness), the same research assistants coded whether each response included expressions of love and kindness (e.g., telling the person who is suffering that they are loved and that people care about them). The coders coded whether the category was present or not (“1” = present; “0” = not present). Interrater reliability between the three coders was high (the percentage of agreement ranged from 68.47% to 81.98%, with an average of 73.57%).

#### 2.2.5. Demographics Questionnaire

Participants completed a demographics questionnaire that asked about their gender, ethnicity, age, socioeconomic status, and the city and country in which they were born and raised.

## 3. Results

### 3.1. Do U.S. Americans Want to Avoid Feeling Negative More than Mexicans Do?

To test Hypothesis 1, we conducted a one-way between-subjects ANOVA to examine the effect of culture (Mexican or U.S. American) on the mean-deviated ANA score. As predicted, U.S. Americans wanted to avoid feeling negative (*M* = 1.16, *SD* = 0.59) more compared to Mexicans (*M* = 0.86, *SD* = 0.56), *F*(1, 111) = 8.03, *p* < 0.01, *η_p_*^2^ = 0.07. To examine the independent effect of ANA from actual negative affect, in line with past research (e.g., Koopmann-Holm & Tsai, 2014), we ran a one-way between-subjects ANCOVA on the mean-deviated ANA score with culture as the independent variable and actual negative affect as covariate. The previously reported findings held. U.S. Americans wanted to avoid feeling negative (estimated marginal *M* = 1.16, *SE* = 0.08) more compared to Mexicans (estimated marginal *M* = 0.86, *SE* = 0.08), *F*(1, 110) = 7.39, *p* < 0.01, *η_p_*^2^ = 0.06.

We also examined cultural differences in ideal positive affect, actual positive affect, and actual negative affect to test whether our findings were specific to ANA. Mexicans ideally wanted to feel positive less (*M* = 1.36, *SD* = 0.47) than did U.S. Americans (*M* = 1.58, *SD* = 0.54), *F*(1, 111) = 5.12, *p* < 0.05, *η_p_*^2^ = 0.04. We did not find cultural differences between Mexicans and U.S. Americans in their actual positive affect (*p* > 0.25) and actual negative affect (*p* > 0.10).

Because of these differences in ideal positive affect, we wanted to examine whether the cultural differences in ANA held after controlling for ideal positive affect. We ran an ANCOVA to examine the effect of culture on ANA, controlling for ideal positive affect. Again, the previously reported finding held. U.S. Americans wanted to avoid feeling negative (estimated marginal *M* = 1.12, *SE* = 0.07) more compared to Mexicans (estimated marginal *M* = 0.91, *SE* = 0.07), *F*(1, 110) = 4.08, *p* < 0.05, *η_p_*^2^ = 0.04.

### 3.2. Do Compassionate Responses Consist of More Emotion Sharing for Mexicans Compared to for U.S. Americans?

To test Hypothesis 2, we conducted a one-way ANOVA on the emotion-sharing score of the open-ended responses (averaged across the three coders) with culture as the independent variable. As predicted, the responses from our Mexican participants focused more on emotion sharing (*M* = 0.16, *SE* = 0.04) compared to the responses from our U.S. American participants (*M* = 0.03, *SE* = 0.04), *F*(1, 109) = 6.94, *p* = 0.01, *η_p_*^2^ = 0.06. We reran this analysis for the expressions of love and kindness score (averaged across the three coders). In line with our reasoning, Mexican (*M* = 0.40, *SE* = 0.05) and U.S. American responses (*M* = 0.41, *SE* = 0.05) did not differ in how much they focused on expressions of love and kindness, *F*(1, 109) = 0.02, *p* = 0.89, suggesting that the cultural differences are specific to emotion sharing.

### 3.3. Do Mental Representations of a Compassionate Face Depict More Sadness and Less Happiness for Mexicans than for U.S. Americans?

**Figure 1:**
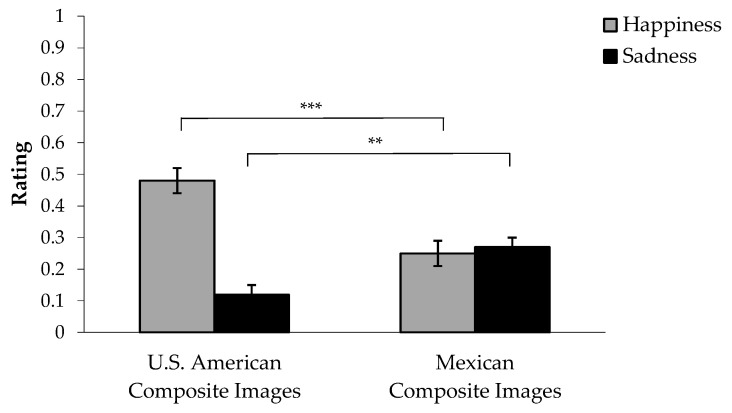
Group differences in the degree of happiness and sadness depicted in the individual composite images. ** *p* < 0.01; *** *p* < 0.001.

To test Hypothesis 3, we used the sadness and happiness manual coding scores (averaged across the three coders) of the individual composite images. We ran a one-way ANOVA to examine the effect of culture on mean sadness and another ANOVA on mean happiness depicted in the composite images. In line with our hypothesis and illustrated in Figure 1, Mexican composite images depicted more sadness (*M* = 0.27, *SE* = 0.03) compared to the U.S. American composite images (*M* = 0.12, *SE* = 0.03), *F*(1, 112) = 9.73, *p* < 0.01, *η_p_*^2^ = 0.08. Additionally, as hypothesized and illustrated in Figure 1, Mexican composite images depicted less happiness (*M* = 0.25, *SE* = 0.04) compared to U.S. American composite images (*M* = 0.48, *SE* = 0.04), *F*(1, 112) = 18.08, *p* < 0.001, *η_p_*^2^ = 0.14. Importantly, while U.S. American composite images depicted statistically significantly more happiness than sadness, *F*(1, 58) = 44.73, *p* < 0.001, for Mexican composite images, we did not find a statistically significant difference between how much happiness and sadness they depicted, *F*(1, 54) = 0.14, *p* = 0.71, suggesting that Mexican composite images depicted equal amounts of happiness and sadness.

### 3.4. Do ANA and Placing Importance on Emotion Sharing for Compassion Sequentially Mediate the Cultural Differences in Conceptualizations of a Compassionate Face?

**Figure 2:**
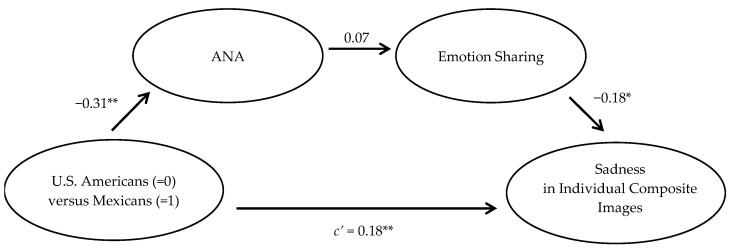
Sequential mediational model where culture influences Avoided Negative Affect (ANA), which then affects conceptualizing a compassionate response as emotion sharing, ultimately shaping the level of sadness depicted in the individual composite images of a compassionate face. * *p* < 0.05; ** *p* < 0.01.

We tested the indirect effect of culture on the amount of sadness depicted in the individual composite images through ANA and then through emotion sharing as an important aspect of a compassionate response (Hypothesis 4) in a sequential mediation model using Hayes’ PROCESS Macro Version 4.1 (Model 6) for SPSS Version 30 (Hayes, 2017). Culture (U.S. Americans = 0, Mexicans = 1) was entered as the independent variable, and the mean sadness score (averaged across the three coders) of the individual composite images was entered as the outcome variable. ANA was entered as the first mediator, and the emotion sharing score of the open-ended responses (averaged across the three coders) was entered as the second. In line with past research (e.g., Koopmann-Holm & Tsai, 2014), which controls for actual negative affect when examining ANA, actual negative affect was entered as statistical control. As predicted, the sequential mediation through ANA and then through emotion sharing was statistically significant. It was estimated to lie between 0.0001^2^ and 0.0126 with a 95% confidence interval. The direct effect of culture on the conceptualization of a compassionate face as depicting sadness was still statistically significant, *B* = 0.18 (95% CI: 0.07 to 0.28), *SE* = 0.05, *t*(106) = 3.32, *p* < 0.01. This suggests that ANA and emotion sharing partially sequentially mediate the cultural differences in how much people consider a sad face to be a compassionate face. The sequential mediation model is depicted in Figure 2^3^.

We reran this sequential mediation model but replaced the outcome variable sadness with the mean happiness score (averaged across the three coders) of the individual composite images. Not in line with our predictions, the sequential mediation through ANA and then through emotion sharing was not statistically significant. It was estimated to lie between −0.0146 and 0.0019 with a 95% confidence interval. The direct effect of culture on the conceptualization of a compassionate face as happy was still statistically significant, *B* = −0.27 (95% CI: −0.39 to −0.15), *SE* = 0.06, *t*(106) = −4.40, *p* < 0.001. Hence, ANA and emotion sharing did not sequentially mediate the cultural differences in how much people consider a slight smile to be part of a compassionate face^4^.

To examine whether the sequential mediation finding including culture, ANA, emotion sharing, and how much people consider sadness to be part of a compassionate face was specific to emotion sharing, we reran the two sequential mediation models as described above, but this time, we replaced emotion sharing with the expressing love and kindness score (averaged across the three coders). None of the indirect effects were statistically significant, suggesting that cultural differences in how much people consider sadness to be part of a compassionate face can be explained by ANA and then specifically through considering emotion sharing as an important aspect of compassionate responses, not through considering expressions of love and kindness as important aspects of compassionate responses.

## 4. Discussion

The current study examined cultural differences between U.S. Americans and Mexicans in their conceptualization of compassionate faces, the degree to which they consider emotion sharing to be part of compassionate responses, and ANA. Given the underrepresentation of Mexicans in psychological research, conducting this study was essential to address this gap and enhance the generalizability of previous findings (e.g., Larco et al., 2024). In support of our hypotheses, we found key cultural differences in how compassion is conceptualized and, using mediational analyses, found support for a sequential mediation model explaining these cultural differences.

### 4.1. Cultural Differences in ANA

As predicted, U.S. Americans wanted to avoid feeling negative more compared to Mexicans. This finding aligns with prior research indicating that people in U.S. American culture want to avoid negative emotions more so than people in many other cultural contexts, such as China, Ecuador, and Germany (e.g., Koopmann-Holm et al., 2021; Larco et al., 2024; Seow et al., 2025). The finding that Mexicans want to avoid feeling negative less may reflect a cultural orientation that is more accepting of negative emotions, particularly in relational contexts, which could be influenced by the collectivistic nature of Mexican culture, where emotional experiences, including negative ones, are often shared and integrated into social interactions (Tousignant, 1984; Tousignant & Maldonado, 1989). While social connection can be achieved via sharing positive emotions, in certain contexts (such as when someone is suffering), sharing negative emotions might be the more socially engaging response.

Thus, while people in Latin America value positive emotions, they are not motivated to avoid negative emotions as much as U.S. Americans are. This finding remained consistent when comparing samples from Ecuador (Larco et al., 2024) and Mexico (in the present study) to U.S. American samples, indicating its robustness across cultural contexts. This aligns with broader patterns in Latin American cultural values, which emphasize the acceptance of negative emotions (e.g., Górriz et al., 2021a) alongside the appreciation of positive emotions (e.g., Senft et al., 2021), in contrast to the stronger preference for positivity and stronger desire to avoid negativity observed in U.S. American contexts (e.g., Koopmann-Holm & Tsai, 2014).

Interestingly, we found that Mexicans ideally wanted to feel positive less than did U.S. Americans, which is in line with the findings that Ecuadorians ideally wanted to feel positive less than did U.S. Americans (Larco et al., 2024) and that European Americans may value positive emotions even more than do Latin Americans (Senft et al., 2021). Taken together, while Latin Americans want to feel positive, they value positivity to a less extreme degree compared to U.S. Americans. Certainly, in our research, we did not differentiate between positive socially engaging and disengaging emotions, so future research should examine cultural differences in ideal positive affect separately for these different kinds of positive states.

As pointed out above, Latin Americans also want to avoid feeling negative to a less extreme degree compared to U.S. Americans. Interestingly, for U.S. Americans, an excessive desire to feel good and to avoid negative emotions may have unintended effects. Studies suggest that striving for positivity can actually reduce positive feelings (e.g., Mauss et al., 2011), while wanting to avoid negative emotions can make individuals feel even more negative when faced with distressing stimuli (e.g., Koopmann-Holm et al., 2020a). While we did not find statistically significant cultural differences in actual positive and actual negative affect in the present study, past work has found that Ecuadorians actually feel more positive and less negative than U.S. Americans do over the course of a typical week (Larco et al., 2024), which aligns with other work (e.g., Holloway et al., 2009). While not statistically significant, the means for actual positive and actual negative affect in the present study are in line with this past work, suggesting that high levels of ANA and ideal positive affect might compromise emotional well-being.

### 4.2. Cultural Differences in Emotion Sharing as Part of Compassionate Responses

As predicted, Mexican participants placed more importance on emotion sharing in their conceptualizations of a compassionate response compared to U.S. Americans. Emotion sharing involves actively engaging with others’ feelings, which seems to be a key component of compassion in collectivistic cultures where interdependence and relational connectedness are prioritized (Larco et al., 2024). The higher importance of emotion sharing for Mexicans compared to U.S. Americans suggests that compassionate responses are conceptualized as deeply relational and based on shared emotional experiences. This finding is consistent with past research, which found similar patterns in an Ecuadorian sample (Larco et al., 2024) and work showing that Latin Americans engage in more emotion sharing when they feel compassion than people in other cultures (Chang et al., 2020). Finally, this finding also aligns with Latin Americans expressing more positive and negative socially engaging versus disengaging emotions (Salvador et al., 2024). Taken together, these findings support the idea that Latin American cultures emphasize interpersonal emotional connectedness as a fundamental aspect of compassion.

### 4.3. Cultural Differences in What People Consider to Be a Compassionate Face

Our third hypothesis focused on how U.S. Americans and Mexicans differ in their mental representations of a compassionate face. As predicted, we found that Mexican composite faces depicted more sadness and less happiness compared to U.S. American composite faces. We found these differences despite the fact that reverse correlation images include a significant amount of noise (Kevane & Koopmann-Holm, 2021). Mexicans seem to associate compassion more strongly with sadness and less with happiness compared to U.S. Americans. Again, this finding replicates the finding by Larco et al. (2024). In collectivistic cultures like Mexico and Ecuador, compassion seems to be viewed as an emotional response to suffering, where expressions of sadness signal empathy and understanding of another’s pain. In contrast, U.S. American conceptualizations of compassion seem to be viewed as an emotional response that focuses on the alleviation of suffering by focusing on the positive. This finding replicates previous research. U.S. Americans consistently visualize a compassionate face with a slight smile, regardless of variations in the base face used for U.S. American participants in different studies (e.g., Koopmann-Holm et al., 2021; Larco et al., 2024; Seow et al., 2025). Hence, the U.S. American conceptualization of a compassionate face replicates despite methodological differences. Moreover, converging evidence suggests that U.S. Americans conceptualize compassion in a more positive way compared to people in many other cultural contexts (e.g., Germany, China, Ecuador, and Mexico; e.g., Koopmann-Holm et al., 2021; Larco et al., 2024; Seow et al., 2025). This suggests that U.S.-based research on compassion, shaped by U.S. American participants’ and researchers’ culture-specific views of a compassionate face, may not directly apply to cultures that conceptualize compassion differently.

Interestingly, again in line with Larco et al. (2024), while for U.S. Americans, a compassionate face depicted more happiness than sadness, for Mexicans, a compassionate face depicted happiness and sadness to similar degrees. As this conceptualization of a compassionate face as depicting similar levels of happiness and sadness had only been described in one previous study (Larco et al., 2024), it was important to examine whether the same conceptualization would be found in another Latin American cultural context. We did. Our almost exact replication of findings reported in previous research (Larco et al., 2024) underlines the importance of recognizing a third form of compassion, namely compassion that focuses on both the positive *and* the negative. The finding that Latin Americans also express positivity when being compassionate is in line with other research suggesting that Latin Americans express positive, socially engaging emotions in negative situations affecting others, which call for compassion (Salvador et al., 2024). Our finding that U.S. American conceptualizations of compassion depict even more happiness than Latin American conceptualizations of compassion is *not* inconsistent with past work suggesting that Latin Americans express positive socially engaging emotions more in negative situations affecting others compared to European Americans (Salvador et al., 2024), as that work examined friendly feelings and feelings of closeness (positive socially engaging emotions), whereas we focused on happiness. Taken together, for Mexicans and Ecuadorians, and possibly also for people in other Latin American cultural contexts, compassion may not necessarily be defined by a single emotional valence but instead reflects a more nuanced, balanced emotional state that integrates both sadness and happiness to similar degrees, possibly to promote and sustain social relationships (see Salvador et al., 2024).

### 4.4. Sequential Mediation: ANA and Emotion Sharing Explain Cultural Differences in Amounts of Sadness in Conceptualizations of a Compassionate Face

Our final hypothesis examined whether ANA and emotion sharing sequentially mediated the cultural differences in how participants conceptualized a compassionate face. The findings provided partial support for this mediation model. ANA and emotion sharing mediated the relationship between culture and the amount of sadness in conceptualizations of compassionate faces. Specifically, because of U.S. Americans’ strong desire to avoid negative emotions, for them, a compassionate response includes less emotion sharing, and therefore, for them, a compassionate face depicts less sadness. In contrast, Mexicans’ lower ANA is associated with a greater emphasis on emotion sharing in a compassionate response, which relates to their conceptualization of compassion as more connected to sadness. As previous research has only conducted exploratory analyses examining this model, it was important to examine whether these associations would replicate in new samples and even in a sample from a different Latin American cultural context (i.e., Mexico). Still, our study uses a cross-sectional design. While examining mediation using such a design is a valuable first step (Murray et al., 2021), future research should employ longitudinal and experimental designs to determine true mediation.

However, not in line with our predictions, ANA and emotion sharing did not sequentially mediate the cultural differences in how much people consider happiness to be depicted in compassionate faces, suggesting that conceptualizations of compassionate faces as expressing a kind smile might be less tied to ANA and emotion sharing than conceptualizations of compassionate faces as mirroring suffering. A possible explanation for why ANA (and emotion sharing) mediated differences in sadness but not happiness is that the ‘avoided affect mismatch‘ might be even more specific than previously thought: When faced with negativity such as another person’s suffering, ANA might not shape the whole response (i.e., the amount of happiness and sadness depicted in a compassionate face), but rather only the part of the response that focuses on the negative (the amount of sadness depicted in a compassionate face in the present study). In line with this reasoning, Koopmann-Holm and Tsai (2014) as well as Koopmann-Holm et al. (2021) found that ANA partly mediated cultural differences in how people rated sympathy cards that focused on the negative, not the positive, suggesting that ANA might be related specifically to responses that focus on what people want to avoid. In fact, Seow et al. (2025) also found that ANA only partially mediated Chinese–U.S. American differences in the amount of sadness (*not* happiness) included in mental representations of a compassionate face. The fact that the present study found the same pattern supports the specificity of the ‘avoided affect mismatch’. Despite this possible specificity, the present study replicates past findings suggesting the importance of emotional goals for how people conceptualize compassion.

The fact that expressions of love and kindness did not differ between cultures and did not mediate the cultural differences in conceptualizations of compassionate faces suggests that emotion sharing in particular is key to understanding how culture shapes conceptualizations of compassionate faces. While expressions of love and kindness seem to be important for compassion in both Latin American and U.S. American cultural contexts, whether or not people consider emotion sharing to be an important aspect of compassion differs across cultures.

### 4.5. Implications and Future Research

These findings contribute to the growing body of research on cultural differences in ANA and compassion (e.g., Koopmann-Holm et al., 2021; Larco et al., 2024; Seow et al., 2025). The results suggest that interventions or practices aimed at cultivating compassion may need to be culturally tailored, as individuals from different cultures may have fundamentally different ways of expressing and interpreting compassionate behaviors. This work has important implications for culturally sensitive counseling and therapy. Therapists can only be truly compassionate when they understand their client’s background and what their client considers to be compassionate. Furthermore, our work demonstrates the importance of considering diversity in the conceptualization of a construct, such as compassion. Our findings suggest that it is important to investigate what is regarded as compassionate in specific cultural contexts before examining the percentages of people in these cultures who engage in certain predefined “compassionate” behaviors. The same behaviors might not be considered equally compassionate in these contexts, and without realizing this, findings will have limited applicability.

Future research could further investigate how these cultural differences in the conceptualization of compassion manifest in other contexts, such as in actual compassionate behavior or in helping interactions. Moreover, examining how these differences in conceptualizing compassion impact cross-cultural interactions could provide valuable insights for improving intercultural communication. Considering the emphasis on reciprocity and interpersonal relatedness in Latin American cultural contexts, future research should incorporate additional affective states into the AVI that vary in their degree of social engagement or disengagement. Moreover, integrating culturally specific terms such as *simpatía* or *pena* into the AVI could enhance its cultural relevance and applicability.

Exploring why people in different cultural contexts differ in ANA will be important for future research as well. Past work has examined a possible basis for U.S.–German cultural differences in ANA, namely the “voluntary settlement hypothesis” (e.g., Kitayama et al., 2006; Kitayama et al., 2009): in the face of famine and hardship, those who left their homelands in Europe to realize their dream of a brighter future became the early settlers of the United States. It is possible that U.S. Americans want to avoid feeling negative more than their German counterparts partly because their European American ancestors went to the new promised land to escape hardship and, in doing so, created a culture in which people want to avoid feeling negative. The ancestors of today’s Germans, in contrast, who stayed in Europe and endured the hardship, might have created or might have already been part of a culture in which people accept the negative more. The high level of ANA among U.S. Americans might stem from “frontier spirit” values, such as mastering one’s environment and escaping negative circumstances, which European Americans endorse more than Germans and which can partly explain U.S. American–German differences in ANA (Koopmann-Holm & Tsai, 2014). Future research could examine cultural values that can explain Latin American–U.S. American cultural differences in ANA.

### 4.6. Limitations

One limitation of the present study is the reliance on student samples from two specific cultural contexts, each of which received different forms of compensation for participation. This sampling approach may constrain the generalizability of the findings to broader populations. Additionally, operationalizing culture as ‘country’ means that important variations within these groups were not explored (e.g., differences between ethnic subgroups within the U.S. or Mexico). Future studies could address these limitations by including more diverse samples and examining the role of additional cultural contexts in shaping ANA and compassion. It would be interesting to look at how subcultures in Mexico, like the indigenous and more rural populations in this country, differ from the population we sampled in the present study in terms of ANA and conceptualizations of compassion. Another limitation of this study is the use of static images to examine conceptualizations of a compassionate face. Future research should explore cultural differences in dynamic compassionate responses and investigate whether factors such as gender and ethnicity of the base face influence these conceptualizations.

Despite these limitations, this study was the first to investigate ANA and conceptualizations of compassion in Mexico. Given the underrepresentation of Mexicans in psychological research, these findings are valuable and hold important implications for cross-cultural counseling. Our findings suggest that there is no “one-size-fits-all” approach in expressing and understanding distinct compassionate responses.

### 4.7. Conclusions

Our study, using multiple methods such as reverse correlation, coding of open-ended responses, and self-report measures, offers valuable insights into how and why U.S. Americans and Mexicans differ in their conceptualizations of compassion. Understanding such cultural differences could foster a genuine understanding of people from different cultural contexts, which includes setting aside one’s own preconceptions of and assumptions about compassion. Through this understanding of people’s differences, true compassion can emerge. When people fully grasp the unique experiences, values, and perspectives of other people, societies can become truly multicultural, where respecting and celebrating the diversity of its members is a key characteristic.

## Data Availability

The data set and syntax are available via the following link: https://doi.org/10.6084/m9.figshare.29142932.

## Notes

1 We used the same materials as did Larco et al. (2024).

2 While this confidence interval does not mathematically include zero, it includes a number that is close to zero. Caution should be used when interpreting indirect effects that just exclude zero (Götz et al., 2021). However, the present study replicates the findings reported in Larco et al. (2024). We were able to not only replicate the cultural differences in ANA, emotion sharing, and sadness and happiness depicted in compassionate faces, but also the indirect effect in this sequential mediation analysis.

3 We also examined the other indirect effects in the model. The indirect effect of ANA alone was not statistically significant. It was estimated to lie between −0.075 and 0.014 with 95% confidence interval using Hayes’ (2017) bootstrapping macro with 10,000 bootstrap samples. The indirect effect of emotion sharing alone was statistically significant. It was estimated to lie between −0.067 and −0.005 with 95% confidence interval.

4 We also examined the other indirect effects in the model. The indirect effect of ANA alone was not statistically significant. It was estimated to lie between −0.019 and 0.049 with 95% confidence interval. The indirect effect of emotion sharing alone was also not statistically significant. It was estimated to lie between −0.014 and 0.078 with 95% confidence interval.
